# Enzymes in Fish and Seafood Processing

**DOI:** 10.3389/fbioe.2016.00059

**Published:** 2016-07-07

**Authors:** Pedro Fernandes

**Affiliations:** ^1^Department of Bioengineering, Institute for Biotechnology and Bioengineering, Instituto Superior Técnico, Universidade de Lisboa, Lisbon, Portugal; ^2^Faculdade de Engenharia, Universidade Lusófona de Humanidades e Tecnologias, Lisbon, Portugal

**Keywords:** biocatalysis, biosensors, proteases, transglutaminases, lipases, oxidases

## Abstract

Enzymes have been used for the production and processing of fish and seafood for several centuries in an empirical manner. In recent decades, a growing trend toward a rational and controlled application of enzymes for such goals has emerged. Underlying such pattern are, among others, the increasingly wider array of enzyme activities and enzyme sources, improved enzyme formulations, and enhanced requirements for cost-effective and environmentally friendly processes. The better use of enzyme action in fish- and seafood-related application has had a significant impact on fish-related industry. Thus, new products have surfaced, product quality has improved, more sustainable processes have been developed, and innovative and reliable analytical techniques have been implemented. Recent development in these fields are presented and discussed, and prospective developments are suggested.

## Introduction

**Figure 1:**
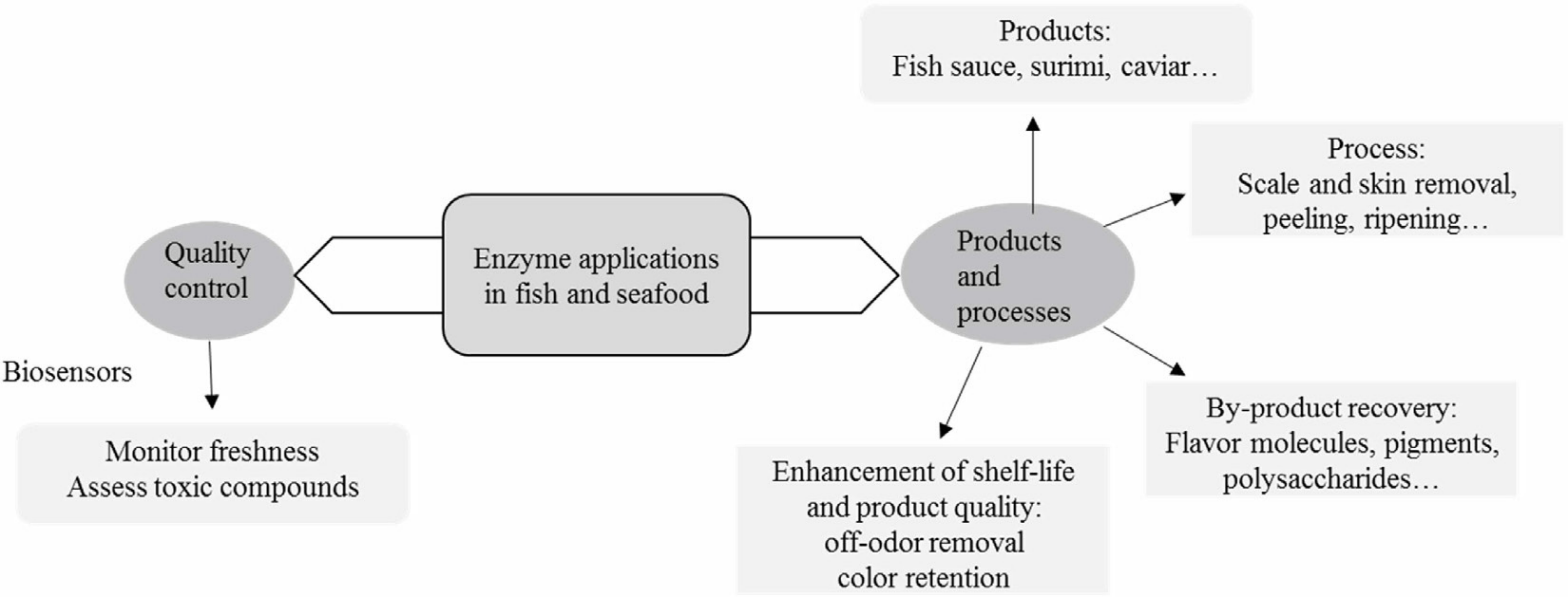
**A schematic overview of enzyme applications in fish and seafood processing**.

Enzymes are key tools in biotechnology and related areas because of their catalytic nature (Fraatz et al., 2014; Jemli et al., 2016). Accordingly, they have been extensively used in food production and processing for centuries, albeit in a rather empirical manner, which has been superseded by a rational approach in the last decades (Whitaker, 1994; Whitaker et al., 2002; Fraatz et al., 2014). In recent years, the focus has been on technical and scientific issues (enzyme formulations, molecular improvement of enzyme, screening for new/improved enzymes through traditional and metagenomics approaches, process improvement) as well as on legal and regulatory matters (definition of enzymes and technological purposes, procedures for safety assessment, harmonization of regulations, among others), all of these abridging the food industry (Fraatz et al., 2014; Li and Cirino, 2014; Alma’abadi et al., 2015; Jemli et al., 2016). Within this latter area, fish and seafood comprise a significant market (Morrissey and DeWitt, 2014), where enzyme action plays an effective role. In particular, and somehow not surprisingly, the use of enzymes from the marine environment has gradually been emerging as a relevant tool for fish and seafood processing (Diaz-López and García-Carreño, 2000; Shahidi and Janak Kamil, 2001; Venugopal, 2005; Sana, 2015), although this is sometimes overshadowed by other applications in food production and processing, e.g., bakery, beverages, and starch processing (Fraatz et al., 2014). This paper aims to provide an overview on the current status on the relevant uses of enzymes for fish and seafood processing and analysis. These are illustrated in Figure 1.

## Enzyme Sources

When considering the enzymatic processing of fish and seafood, the role of both endogenous and added enzymes has to be considered. In the latter case, the enzymes used are from mammalian, plant, or microbial sources. Ease of manipulation and cultivation of the latter makes them the preferred source of enzymes. These are typically from terrestrial organisms, yet given the wide pool of marine microorganisms, the trend toward the use of these as enzyme sources has been increasing (Trincone, 2011, 2013). In particular, they are often adapted so as to display high activity at relatively low temperatures, unlike many of enzymes from terrestrial sources, thereby making them more effective in many processes that require often a low-temperature environment (Simpson, 2012).

## Process Applications

Traditional use of enzymes in seafood processing involves the use of proteases, namely bacillolysin (Neutrase^®^), ficin, papain, pepsin, subtilisin (Alcalase^®^), trypsin, and a mixture of bacillolysin and subtilisin (Protamex^®^), of both endogenous and exogenous nature, for descaling and deskinning, peeling of shrimp, production of caviar and fish sauce, recovery of diverse molecules, and tenderization of squid, as reviewed by several authors (Haard and Simpson, 1994; Vilhelmsson, 1997; Diaz-López and García-Carreño, 2000; Suresh et al., 2015).

### Proteases

Proteases are widely used in fish and seafood processing (Diaz-López and García-Carreño, 2000; Suresh et al., 2015), covering a wide array of applications.

#### Deskinning and Descaling

Deskinning involves the removal of fish skin without causing damage to the flesh, a process currently performed by rough mechanical procedures, imparting considerable risk of damaging the flesh and producing excessive waste. Moreover, enzymatic deskinning can improve the edible yield. Several specific applications have been implemented, specifically for processing herring, pollock, squid, skate, shrimp shells, and tuna, occasionally combined with physical treatment (Haard and Simpson, 1994; Rasika et al., 2013). Several of these methods involve the use of enzymes from marine organisms, for example, acid proteases from cod viscera for herring, protease extracts from minced arrowtooth flounder for pollock, and enzymes from squid for squid itself (Simpson, 2012). Recently, commercial proteases (Proleather FG-F^®^ and Protease N^®^) and collagenase (CLS1^®^) were tested for the deskinning of catfish nuggets. Proleather FG-F^®^ proved effective, and operational conditions (enzyme concentration, time, and temperature of incubation) were identified that optimized removal of the peritoneal membrane (Kim et al., 2014). Descaling can also be performed by mechanical methods, but again it is a harsh treatment and may result in tearing of the skin and lower filet yield. Thus, the milder enzyme approach is favored, particularly if mixtures of fish digestive proteases that enable operation at low temperatures, are used (Svenning et al., 1993; Gildberg et al., 2000; Gildberg, 2004). This approach has been assessed for scale removal of haddock and redfish (Haard and Simpson, 1994) in Japanese sashimi restaurants and fresh-fish markets (Simpson, 2012).

Proteases have also been used for the removal of raw meat from the head-shell of crustaceans, by immersing the latter in an enzyme solution (Gallant et al., 2001), although the reliability of the method has been questioned (Jabbour and Hognason, 2007). Early efforts for shrimp peeling and de-veining and for shucking clams have been reported, involving a mixture of ficin and amylase in the former case and ficin, amylases, and cellulases in the latter (Venugopal et al., 2000).

#### Fish Protein Hydrolyzate

One of the major established applications is the production of fish protein hydrolyzates (FPHs). FPH is the result of the enzymatic (*endo*- and/or *exo*-peptidases) or chemical hydrolysis of protein-rich byproduct waste of the fish processing industry, such as bones, head, liver, skin, trimmings, and viscera of fish flesh and of minces, leading to peptides with 2–20 amino acids, depending on the enzymes used, the fish used as source, the time of incubation, and concomitantly the degree of hydrolysis (*D*_h_), defined as the ratio of the number of broken peptide bonds (*p*) to the total number of peptide bonds per mass unit (*p_tot_*).

$$D_{\text{h}}\;=\;\frac{p}{p_{\text{tot}}}\text{x100}$$

Thus, a free α-amino group is formed for each hydrolyzed peptide bond (Nguyen et al., 2011; Chalamaiah et al., 2012; Benjakul et al., 2014; He et al., 2015; Suresh et al., 2015).

Traditional FPH hydrolysis was promoted either by acid or alkali action. Acid hydrolysis involves the use of concentrated hydrochloric acid, or occasionally sulfuric acid, operation at high temperatures and pressures, and neutralization of the hydrolyzate. Accordingly, the hydrolyzate contains large amounts of sodium chloride, which impairs its functionality. Moreover, tryptophan, a key amino acid, is destroyed in the process. Alkali hydrolysis also involves relatively high temperatures and concentrated sodium hydroxide. Moreover, during the process, several unwanted reactions occur, which lead to the formation of toxic compounds and impair the functionality of the hydrolyzate (Kristinsson and Rasco, 2000). The enzyme approach, although complex, occurs under mild conditions of temperature, pressure, and pH and involves the use of proteolytic enzymes, typically available at low cost, and deleterious reactions are virtually nonexistant. Hence, this approach is technically and economically attractive (He et al., 2013). FPHs display functional properties of interest for food formulation, namely emulsification and foam-forming ability, gelling activity, protein solubility, oil-binding capability, and water-holding capacity (Kristinsson and Rasco, 2000; Chalamaiah et al., 2010; He et al., 2015). FPHs compare favorably with poultry byproducts and protein hydrolyzate, both obtained by proteolysis with Alcalase, an outcome ascribed to the difference in amino acid composition (Taheri et al., 2013). Moreover, FPHs are envisaged as effective source of proteins for human nutrition, given their balanced amino acid composition and easier gastrointestinal adsorption when compared to free amino acids (Clemente, 2000). FPHs also exhibit antioxidant, antihypertensive, immunomodulatory, and antimicrobial activities, hence their incorporation in nutraceuticals and functional/health foods has recently emerged, as evidenced by the presence of several commercially available products (Chalamaiah et al., 2012; Hu et al., 2015). FPHs have a bitter taste, which is one of the key issues that prevents its dissemination in food products, East Asian condiments, and sauces (Kristinsson and Rasco, 2000). Moreover, excessive hydrolysis is likely to impair some functional properties or cause off-flavors in the final product (Balti et al., 2010). Several approaches for de-bittering have been tested, with different advantages and limitations. Among these, the enzymatic action of exopeptidases and concomitant removal of free amino acids has emerged as the most promising (Sujith and Hymavathi, 2011). Recently, the possibility of using FPHs as cryoprotective agents to preserve frozen fish, as an alternative to commonly used carbohydrate-based agents, was suggested, given the positive results obtained when hydrolyzates from Pacific hake (*Merluccius productus*) were obtained upon proteolysis using either Alcalase or Flavourzyme. FPHs were used for frozen storage of minced cod samples and displayed similar or better cryoprotective properties than a standard sucrose–sorbitol mixture (Cheung et al., 2009).

The production of FPHs typically requires the addition of enzymes from different sources, namely plants (papain, bromelain), microorganisms involving both commercial formulations (Alcalase, Flavourzyme, Neutrase, Protamex) and crude enzyme preparations (orientase, papain, trypsin, thermolysin), and fish digestive enzymes (pepsin, trypsin, chymotrypsin) (Kristinsson and Rasco, 2000; Benjakul et al., 2014; Jridi et al., 2014; Yarnpakdee et al., 2015). The production of FPHs using either endogenous enzymes such as cathepsin L in autocatalytic processes or endogenous enzymes combined with exogenous enzymes has also been performed (Kristinsson and Rasco, 2000; Samaranayaka and Li-Chan, 2008; Ovissipour et al., 2013). Still, processes based only on the use of endogenous enzymes lead to low protein content and recovery (Samaranayaka and Li-Chan, 2008; Ovissipour et al., 2013).

Efforts have been recently made to optimize the process of FPH production and to make better use of wastes in order and obtain products with relevant activity (Table 1). Further details on the use of enzymes for this application can be found in a recent review (Benjakul et al., 2014).

**Table 1:** **Recent developments on the production of FPHs with application in food and feed**.

Goal/summary	*D*_h_ (%)	Reference
Optimization of Alcalase-catalyzed hydrolysis of cobia frame using response surface methodology (RSM). *D*_h_ was maximized with an enzyme concentration of 8.3%, temperature of 58°C, hydrolysis time of 134 min, and pH of 9.4. The hydrolyzate contained 88.8% protein, 0.58% fat, and 5.05% ash	96	Amiza et al. (2014)
Optimization of shrimp waste protein hydrolyzate using Alcalase and RSM. A model equation was developed that correlated temperature, pH, enzyme/substrate ratio, and time with *D*_h_	33	Dey and Dora (2014a)
Use of Alcalase for the production of shrimp waste protein hydrolyzate with antioxidative properties	n.d.	Dey and Dora (2014b)
Optimization of the production of carotenoids and protein hydrolyzate with antioxidative properties through RSM applied to the hydrolysis of shrimp waste using Alcalase. Optimal temperature, enzyme concentration, and time of incubation depended on the targeted product	n.d.	Sowmya et al. (2014)
Assessment of the validity of hydrolyzing the byproducts resulting from processing of tilapia fish into filets using Alcalase. The final product had a high protein content (62.71%), contained 199.15 mg essential amino acids per gram, and displayed high angiotensin converting enzyme inhibitory activity	20	Roslan et al. (2014)
Optimization of the papain-catalyzed hydrolysis of byproducts from catfish filet production. Optimal operational conditions were identified as a temperature of 60°C, pH 5, enzyme concentration4% (w/w), and time of hydrolysis of 48 h	n.d.	Utomo et al. (2014)
Optimization of Neutrase-catalyzed hydrolysis of FPH from fish muscle using RSM, aiming at the highest content of sweet and umami taste amino acids. Optimal temperature, pH, and enzyme/substrate ratio were established as 40.7°C, 7.68, and 0.84%, respectively	17	Shen et al. (2012)
Production of FPH in a batch process at 50 l pilot plant scale, through papain-catalyzed hydrolysis of cod and haddock fish frames. Almost complete hydrolysis could be achieved in 1 h, at 40°C, and 0.5% enzyme/substrate ratio. FPH products were fit for both human and animal consumption	≈ 100	Himonides et al. (2011b)

#### Fish Sauce

Fish sauce is the outcome of enzyme-solubilized and digested fish protein. The preparation is preserved in salt and used as an ingredient and condiment on vegetable dishes. Currently associated with Southeast Asia, fish sauce was quite popular in Roman culture, but since then it has almost vanished from Europe (Gildberg et al., 2000; Tanasupawat and Visessanguan, 2014). Protein hydrolysis takes place by autolysis, mainly involving trypsin and chymotrypsin, alongside cathepsins. As the pH of fish sauce decreases during fermentation, from around 7 to 5, the role of the enzymes in protein digestions is complementary because, while the two former ones are active at pH ~7, the latter are active in acidic environments (Lopetcharat et al., 2001; Turk et al., 2012). The extent of proteolysis is typically characterized by *D*_h_.

The traditional process, which relies solely on autolysis involving fermentation and endogenous enzymes, is quite time consuming because it takes several months up to 3 years for full completion (Lopetcharat et al., 2001; Gildberg et al., 2007; Faisal et al., 2015; Lee et al., 2015). Therefore, the use of exogenous enzymes, such as bromelain, ficin, or papain, as well as the commercial preparations Protamex and particularly Protex 51FP and Neutrase, has been also shown to speed up fish fermentation (Beddows and Ardeshir, 1979; Ooshiro et al., 1981; Chuapoehuk and Raksakulthai, 1992; Aquerreta et al., 2001; Himonides et al., 2011a; Le et al., 2015). The use of exogenous enzymes can increase significantly the pace of fish fermentation (Aquerreta et al., 2001), yet their application should be carefully assessed in order not to tamper with the required functional and organoleptic properties and quality of the final product (Ooshiro et al., 1981; Himonides et al., 2011a; Ghaly et al., 2015). On the other hand, the use of exogenous enzymes can lead to a final product that fulfills the intended role and displays adequate features and even promising nutraceutical properties. This is due to the presence of polyunsaturated fatty acids, lower level of salt, and higher protein content than currently produced fish sauces in Southeast Asia. The preparation obtained with exogenous enzymes was also considered to be more similar to the fish sauce produced in classical Roman age (Aquerreta et al., 2001). Moreover, and besides process conditions, the functional and organoleptic properties and the nature of the final product are noticeably influenced by the starting material, as reported by Gildberg *et al*., who compared fish sauces obtained from several cold-water and tropical species. Thus, not only the overall content of proteins but also the profile of amino acids differs, and possibly that of fatty acids, which is likely the result of the diverse endogenous microbial and enzymatic activity (Gildberg et al., 2007). Different contents of protein and lipidic components were also observed when sardines and anchovy as raw materials were compared (Le et al., 2015). The ratio of salt to fish also conditions enzyme activity, with impact on the characteristics of the final product (Gildberg et al., 2007; Tanasupawat and Visessanguan, 2014; Le et al., 2015). Hence, the detailed characterization of catalytic activity requires chemical, physical, and sensorial evaluation, alongside microbiological analysis (Aquerreta et al., 2001; Gildberg et al., 2007; Tanasupawat and Visessanguan, 2014; Faisal et al., 2015).

#### Ripening

Proteases are also involved in the ripening of salted fish such as herring, anchovy, cod, and salmon, a complex biochemical process mostly characterized by the degradation of muscle proteins with endogenous enzymes, with increase of peptides and free amino acids. The activity of enzymes from the digestive tract, namely chymotrypsin and trypsin, is typically the most significant in the ripening process, although muscle proteases, namely cathepsins, also play a non-negligible role (Sikorski, 2007; Bjørkevoll et al., 2008; Rahaman, 2014).

### Transglutaminase

Transglutaminases (TGAs), protein-glutamine γ-glutamyl-transferase (EC 2.3.2.13), promote acyl transfer reactions. These involve the γ-carboxyamide group of a peptide-bound glutamine residue as acyl donors and several primary amines as acyl acceptors, for example, the ɛ-amino group of lysine. Concomitantly, intra and intermolecular covalent bonds are formed, namely ɛ-(γ-glutamyl)–lysine, resulting in the cross-linking of peptides and proteins and polymerization. When primary amines are absent, water acts as acyl receptor, and the γ-carboxyamide groups of glutamine residues are deaminated into glutamic acid residues (Diaz-López and García-Carreño, 2000; Sikorski, 2007; Zilda, 2014). TGAs are available form mammalian, plant, and microbial sources, the two former being Ca^2+^ dependent (TGase), unlike the latter (microbial TGA; MTGase). This feature, along with the facile and more cost-effective production of enzymes from microbial sources, makes these the source of the diverse commercial formulations of TGAs (Sikorski, 2007; Serafini-Fracassini and Del Duca, 2008; Kieliszek and Misiewicz, 2014; Zilda, 2014).

Given their cross-linking ability, MTGases are used for the modification/improvement of the functional and mechanical properties of fish and seafood products, and therefore are used as binding ingredients for the restructuration of raw meats and in the production of surimi (Mariniello et al., 2008; Kieliszek and Misiewicz, 2014; Zilda, 2014). Also, TGAs are used in formulations of fish meat mince, modification of finfish texture, processing of shark fin, formation of collagen and gelatin bonds, and minimization of drip after thawing (Diaz-López and García-Carreño, 2000; Zilda, 2014; Suresh et al., 2015). Most of these processes involve the use of added MTGases; still, endogenous TGases account for ɛ-(γ-glutamyl)–lysine cross-linking in dried fish, frozen-stored surimi, and the polymerization of myosin heavy chains in the manufacture of kamaboko (Sikorski, 2007). The cost of enzymes may still limit the extention of this strategy to enhance the mechanical properties of these products. Still, their environmentally friendly nature, high activity, and specificity provide a promising alternative or complementary nature to the use of protease inhibitors (which can lead to unwanted changes in color and flavor), phosphates (which have a negative environmental impact), or oxidizing agents (Martín-Sánchez et al., 2009). The application of TGAs in fish and seafood has been reviewed recently (Kieliszek and Misiewicz, 2014; Zilda, 2014; Suresh et al., 2015), yet some illustrative examples of the mentioned applications are given in Table 2.

**Table 2:** **Examples of recent application of MTGs in fish and seafood processing**.

Goal/summary	Ref.
Treatment of extruded fish feed with a commercial MTGase to improve the physical quality of the product	Wolska et al. (2015)
Addition of MTGase to improve the textural properties of Pacific whiting surimi, to allow the production of high-quality fish balls	Yin and Park (2015)
Assessment of the effect of adding MTGase and fish gelatin on the textural, physical, and sensory properties of surimi from threadfin beam	Kaewudom et al. (2013)
Use of MTGase combined with cold gelation technology to obtain different raw products from minced and/or chopped fish muscle	Moreno et al. (2013)
Improvement of the film-forming properties of Channel Catfish (*Ictalurus punctatus*) skin gelatin by cross-linking with a commercial MTGase preparation, Activa^®^	Oh (2012)
Improvement of rheological and film-forming properties of fish gelatin using Activa^®^	Liu et al. (2011)
Optimizations of MTGase concentration for the production of fish restructured boneless filet from white croacker (*Micropogonias furnieri*)	Gonçalves and Passos (2010)

### Lipases and Miscellaneous Process Applications

Lipases, triacylglycerol acylhydrolases (EC 3.1.1.3), promote the hydrolysis of tri-, di-, and monoglycerides to glycerol and fatty acids, in the presence of excess water, while in water-limiting conditions they promote ester synthesis. They often express other activities, namely phospholipase or other esterase type of activity, all of which have acknowledged industrial relevance (Venugopal et al., 2000; Verma et al., 2012). Lipases are of particular interest for the isolation of oil and fats from seafood byproducts as well as in the preparation of ω-3-poly-unsaturated fatty acids (ω-PUFAs) and enriched marine oils, given the nutritional value of these compounds (Chen et al., 2012; Walker et al., 2015). The enrichment performed chemically requires temperature and pH conditions harmful for the labile substrates, and hence the mild conditions required by lipases to promote transesterification are favored (Diaz-López and García-Carreño, 2000). Examples of applications can be found in a recent published review (Chaurasia et al., 2016).

Other applications of enzymes in fish and seafood processing include the production of caviar, the recovery of chitin, collagen, flavor molecules, minerals, and pigments from seafood byproducts, the removal of unwanted odors, and the improvement of shelf-life and color retention (Suresh et al., 2015).

Proteolytic enzymes, namely pepsins, have been used as alternative to mechanical and manual methods in the production of caviar. Extraction of caviar from roe sacs by enzymes minimizes damage to the eggs and results in a product free from connective tissue, as compared to the other methods, thereby leading to higher process yields (Martin et al., 2000; Venugopal, 2009).

Chitin, a linear heterogeneous polysaccharide of *N*-acetyl-d-glucosamine and d-glucosamine, linked by β(1,4) glycosidic bonds, present in crustaceans and mollusks, is the most abundant and renewable polysaccharide on Earth, next to cellulose (Gortari and Hours, 2013). Overall, 10 billion tons of chitin is produced yearly (Zargar et al., 2015). Given its environmental friendliness, biocompatibility, biodegradability, and the relative ease of functionalization, chitin has a wide array of applications. These include food and feed, where chitin can be used as antioxidant, emulsifier, and thickening agent and for clarification of fruit juices, stabilization of color, reduction of lipid adsorption, and the preparation of dietary fibers. In addition, chitin is widely used in biomedical, pharmaceutical, tissue engineering, cosmetics, and wastewater sectors (Zargar et al., 2015). For this byproduct of the crustacean processing industry to be of use, the removal of minerals (demineralization), proteins (deproteinization), and pigments (decolorization) is required (Suresh et al., 2015; Zargar et al., 2015). The conventional process for chitin recovery and purification requires the use of strong alkali and acid solutions and relatively high temperatures. This involves an energy-consuming and environmentally hazardous process and leads to a product of relatively low quality. Moreover, the alkali-based deproteinization process prevents the use of the protein in animal feed (Synowiecki and Al-Khateeb, 2000; Gortari and Hours, 2013; Younes and Rinaudo, 2015; Zargar et al., 2015). Several proteolytic enzymes have been used for protein removal from chitin, such as Alcalase, pancreatine, papain, pepsin, and trypsin. Still, and despite enzyme screening and operational condition optimization, the enzymatic process is less effective than the conventional chemical method, as 5%–10% residual protein remains attached to the purified chitin when the former method is used (Younes and Rinaudo, 2015). In order to overcome this limitation, the use of a mild alkali treatment upon enzymatic proteolysis has been suggested (Younes and Rinaudo, 2015). Moreover, it has been reported that the order of demineralization and deproteinization is largely irrelevant in the yield and quality of chitin purification when chemical methods are used, whereas the presence of minerals may hamper the access of proteolytic enzymes to the substrate (Gortari and Hours, 2013; Younes and Rinaudo, 2015). This may be the reason underlying the data reported by Valdez-Peña and coworkers (Valdez-Pena et al., 2010). These authors reported an ecofriendly process for purifying chitin, with a sequential combination of enzymatic deproteinization and microwave irradiation for demineralization, where despite screening for enzyme activity, the residual protein was roughly half of the initial value (Valdez-Pena et al., 2010). Implementation of enzymatic deproteinization at the commercial scale is also limited by the cost of commercially available enzymes. An alternative approach, eventually more cost effective, involves the use of crude protease preparations from supernatants of microbial fermentations (Paul et al., 2015).

Collagen is a fibrous protein found in animal skin, bone, and connective tissue, accounting for about 30% of total protein content (Pal and Suresh, 2016). Collagen is widely used in the food and beverages industry as antioxidants, emulsifiers, thickeners, and preservatives, but also as edible films and coatings. Moreover, collagen is also used in biomedical, pharmaceutical, tissue engineering, and cosmetics areas (Benjakul et al., 2012; Hashim et al., 2015; Pal and Suresh, 2016). The outbreak of bovine spongiform encephalopathy and bird flu resulted in an increasing demand for collagen from fish, where it can be extracted from the skin, scale, swim bladder, fins, and bones (Benjakul et al., 2012; Pal and Suresh, 2016). Extraction of collagen must be performed at a temperature of ~ 4°C to minimize collagen degradation. Pepsin is the most common enzyme used for collagen extraction, occasionally used together with acetic acid. This enzymatic method displays particular features that are of interest, namely the hydrolysis of non-collagenous proteins, the hydrolysis of the telopeptides of collagen, enhancing its solubility in acid and concomitantly the extraction yield, and simultaneously reducing the antigenicity caused by telopeptides. Despite these advantages, acid extraction is by far the most widely used method for collagen extraction, possibly because of it low cost and ease of implementation (Benjakul et al., 2012; Hashim et al., 2015; Pal and Suresh, 2016).

The recovery of flavor molecules from seafood byproducts relies mostly on the use of commercial protease preparations, for example, Flavourzyme (leucyl aminopeptidase) and Protamex (Suresh and Prabhu, 2013). More recently, the effective use of bromelain has also been reported for the recovery of seafood-like flavor from byproducts of seaweed (Laohakunjit et al., 2014). Despite the limited availability of technical information, the selection of the most adequate enzyme for flavor recovery from seafood byproducts is largely casuistic and depends on the nature of the raw material (Suresh and Prabhu, 2013). Proteases are also used for the recovery of minerals form seafood byproducts, such as fishbone, which is rich in calcium and phosphorus (Suresh and Prabhu, 2013; Suresh et al., 2015).

Carotenoids and melanin are the major pigments found in the byproducts of seafood processing (Suresh and Prabhu, 2013). Carotenoids are used as additives in feed, to convey skin pigmentation in fish, suggestive of high quality and freshness to the consumer (Suresh and Prabhu, 2013; Malaweera and Wijesundara, 2014). The unstable carotenoids are typically extracted from crustaceous waste using organic solvents, which is environmentally hazardous and requires solvent recycling (Malaweera and Wijesundara, 2014). Enzymatic extraction relies on the use of proteases, mostly trypsin, to recover carotenoids in the form of carotenoproteins (Suresh and Prabhu, 2013). Recently, the use of crude protease extracts from the hepatopancreas of Pacific white shrimp allowed the extraction of carotenoproteins from shrimp waste rich in astaxanthin and displaying significant antioxidant activity. The crude nature of the active enzyme extract may underlie the development of a cost-effective methodology (Senphan et al., 2014).

The improvement of the shelf-life of fishery products depends on the development of strategies that prevent the action of deleterious agents such as endogenous enzymes, microbial contamination, and oxidation of lipid compounds. In alternative or alongside methodologies such as active packaging, controlled-atmosphere packaging, and natural preservatives such as plant materials, the use of glucose oxidase and catalase has been reported to prevent lipid oxidation (Campos et al., 2012; Erkmen, 2012; Siró, 2012; Khalafalla et al., 2015). Glucose oxidase has been also used for color retention in cooked shrimp and crab, as the enzyme prevents the oxidation of carotenoids (Venugopal et al., 2000).

Off-odors and fishy taste, mostly due to the presence of urea in the meat of sharks and rays, have been tackled by the use of materials rich in urease, such as soybean flour (Suresh et al., 2015).

## Analytical Applications

Aquaculture and fish production has grown considerably in the last decades (FAO, 2014), but occasional toxic episodes, typically caused by toxins and involving shellfish, raises public health concerns and presents challenges for marketing those goods (FAO, 2011; Rodríguez et al., 2015). Several methods have therefore been developed for toxin screening, among which are enzyme-based methods such as enzyme-linked immunosorbent assays (ELISA). Briefly, this method involves the immobilization of the target antigen to a solid surface and its subsequent complexation with an antibody linked to an enzyme. The detection is carried out by incubating the enzyme, often peroxidase or alkaline phosphatase (Alp), in the presence of substrate, and assessing the formation of an easily measurable product (Rustad, 2010). Hence, commercially available ELISA kits have been successfully tested for the determination and quantification of antimicrobials (e.g., crystal violet, chloramphenicol, gentamicin, fluoroquinolone enrofloxacin, malachite green, metabolites of furaltadone and furazolidone) in fish from aquaculture, to assess illegal use of the compounds (Jester et al., 2014; Conti et al., 2015). These kits have also proved effective in the detection of toxins [e.g., paralytic shellfish poisoning (PSP), diarrhetic shellfish poisoning, neurotoxic shellfish poisoning, and amnesic shellfish] in shellfish and seafood (Garet et al., 2010; Huazhang et al., 2011; Eberhart et al., 2013; Turner and Goya, 2016) and pesticides in fish (Sapozhnikova et al., 2015). Still, when ELISA method was compared to a phosphatase 2A inhibition assay, the latter displayed more promising results as a screening tool for diarrhetic shellfish toxins, given the sensitivity and low level of false results (Eberhart et al., 2013).

Alongside commercial ELISA kits, researchers have developed setups based on ELISA methods anchored in horseradish peroxidase (Hrp). These have been assayed

(a) for the determination of anisakis larvae in seafood, with lower limits of detection within 5 to 250 parasites per kg of sample, depending on the specific features of the method and of the allergen targeted (Arilla et al., 2008; Xu et al., 2010);
(b) for the detection and quantification of malachite green, a dye with antimicrobial and antiparasitic properties, that has been illicitly used as an antifungal agent in aquaculture. The compound is rapidly metabolized to leucomalachite green, both compounds having putative carcinogenic activity. The setup developed allowed a limit of quantification (LOQ) and a limit of detection (LOD) for mixtures of the two compounds of 0.3 and 0.1 μg/kg_fish_, respectively, which are below the concentration of 2 μg/kg_fish_ imposed by the EU as LOD and 1 μg/kg_fish_ imposed by Canadian legislation as the limiting threshold above which fish is not allowed into the market (European Commission, 2004; Singh et al., 2011);
(c) for the detection of *Vibrio parahaemolyticus*, a pathogen that causes gastroenteritis, in seafood, resulting in a methodology that matched results obtained with a PCR (polymerase chain reaction)-based approach, while requiring less expertise and specialized and costly equipment (Kumar et al., 2011).

A peroxidase-based ELISA for the rapid and sensitive monitoring of PSP toxins in shellfish was recently presented. Data obtained with the new method correlated well with the reference mouse bioassay, but showed higher sensitivity, as the LOD was lower than the reference method. Moreover, when positive and negative results were compared based on the regulatory limit, the peroxidase-based ELISA method displayed a sensitivity of 100% and a specificity of 90% compared to the reference method (Kawatsu et al., 2014).

Once established that they allow for adequate response, LOD, and LOQ, the simplicity, high throughput capability, speed, and the relatively low cost of ELISA methods are competitive advantages when compared to costly, time- and man-power-demanding chromatographic or PCR-based methods. Still, ELISA methods depend heavily on the quality of antibodies, the preparation of which is time consuming. Alternatively, the use of aptamers has emerged. Aptamers are small-molecular-weight, single-stranded DNA or RNA molecules with high affinity and selectivity for proteins, which can be synthesized by chemical methods and are more stable than antibodies. An immunoassay based on aptamers, ELAA (enzyme-linked aptamer assay), was developed for the quantitative detection of *Vibrio parahemolyticus*, a pathogen related to seafood poisoning. The setup involves the use of Hrp immobilized onto gold nanoparticles. In the presence of the pathogen, down to 10 CFU/mL, the enzyme promotes a reaction involving 3,3′,5,5′-tetramethylbenzidine and H_2_O_2_, and an optical signal is produced in a linear manner in a logarithm plot within 10–10^6^ CFU/mL (Wu et al., 2015). Also relying on the aptamer-based approach and on Hrp’s ability to catalyze the formation of colored products out of different substrates, an aptasensor for the detection and quantification of chloramphenicol in fish was developed. The sensor has a linear range 0.05–100 ng_chloramphenicol_/mL, which is within the range of the different methods already available, but displays an LOD of 0.015 ng_chloramphenicol_/mL, which is only surpassed by an electrochemical immunoassay. Moreover, when tested in real fish samples, the detection of chloramphenicol matched that obtained with a standard ELISA test (Miao et al., 2015).

**Figure 2:**
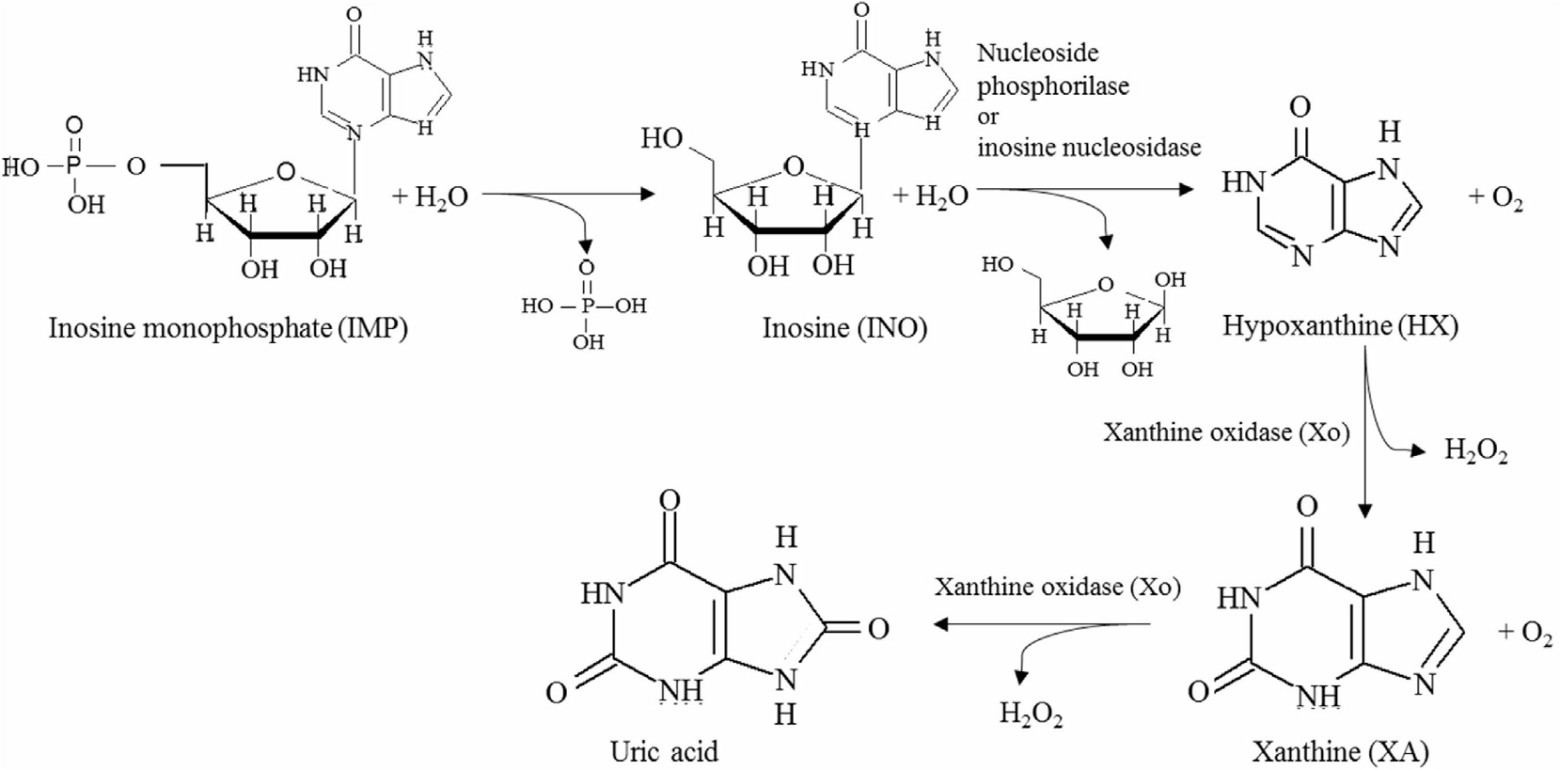
**Relevant steps in the main pathway for nucleotide degradation in fish leading to the production of spoilage indicators, namely inosine and hypoxanthine**.

Enzymatic methods have also been used to establish the freshness of fish and seafood, based on the concentration of nucleotides present (Aristoy et al., 2010). Briefly, once death occurs, adenosine triphosphate (ATP) ceases to be synthesized/regenerated, and it is rapidly decomposed to adenosine monophosphate (AMP) and then to inosine monophosphate (IMP), the former accumulating mostly in crustaceans while the later in fish, where it is responsible for conveying the pleasant fresh flavor (Luong and Male, 1992). IMP spontaneously degrades slowly to inosine (INO), a process slowed by cold environments, and INO is converted to hypoxanthine (HX), which conveys a bitter taste in the presence of either nucleoside phosphorylase (Np) or inosine nucleosidase (In). HX is sequentially oxidized to xanthine (XA) and to uric acid by xanthine oxidase (Xo), with release of hydrogen peroxide in both steps (Nielsen and Nielsen, 2012; Kostić et al., 2015). The key steps of this pathway are illustrated in Figure 2. INO and HX are typically used as indicators of freshness, but given that the variability in the degradation of mononucleotides depends on several factors, e.g., source of material and physical methods of processing, often multiparametric indicators are advised, such as

$$K_{\text{i}}\;=\;\frac{\left[\text{INO}\right]+\left[\text{HX}\right]}{\left[\text{INO}\right]+\left[\text{HX}\right]+\left[\text{IMP}\right]}\text{x100}\;\text{and}\;\text{H}=\;\frac{\left[\text{HX}\right]}{\left[\text{INO}\right]+\left[\text{HX}\right]+\left[\text{IMP}\right]}\text{x100}$$

In any case, the higher the concentration of IMP, the fresher the fish. Enzymatic analysis can be performed with one or more enzymes either in solution or immobilized, in the latter case typically as a biosensor, where the biological component is connected to a physical-chemical transducer and an optoelectronic interface (Aristoy et al., 2010; Thakur and Ragavan, 2013). A review on the materials and principles underlying biosensor assembly and operation targeted for food analysis was published recently (Thakur and Ragavan, 2013).

Biogenic amines (BAs) are nonvolatile, low-molecular-weight organic bases, such as histamine, cadaverine, putrescine, and tyramine, that are formed in foods as the outcome of microbial decarboxylation of the corresponding amino acids or of transamination of aldehydes and ketones by amino acid transaminases (Prester, 2011; Visciano et al., 2012; Zhai et al., 2012). Since BAs are produced by spoilage bacteria, their level can represent the quality of food (Hosseini et al., 2013). The consumption of high amounts of BAs, particularly histamine, can result in food poisoning; hence the maximum content in histamine is regulated. In Europe, these are within 100–200 mg/kg for fish species, and within 200–400 mg/kg for enzyme-processed foods. ELISA kits and histamine-specific enzyme kits, based on the oxidation of histamine by histamine dehydrogenase, are commercially available (Köse et al., 2011; Hungerford and Wu, 2012; Visciano et al., 2012).

The use of free enzymes, preferentially for the quantification of IMP, INO, and HX, involves the sequential use of either 5′-nucleotidase (Nt) or adenosinedeaminase (Ad), Np, and Xo, ultimately resulting in the formation of uric acid and hydrogen peroxide (Luong et al., 1989; Luong and Male, 1992; Cho et al., 1999, 2000). Measurements can be carried out by polarography, as the response of a Clark hydrogen peroxide probe electrode to uric acid and hydrogen peroxide is additive (Luong et al., 1989), or by spectrophotometry (Luong and Male, 1992; Cho et al., 1999, 2000). The combination of Alp, Np, and Xo, conjugated with WST-8, a color developing agent that reacts with hydrogen peroxide allowing spectrophotometric readings, was used for the colorimetric-based quantification of IMP, INO, and HX. Amorphous freeze-dried enzyme formulations in the presence of gelatin and sucrose were prepared, which allowed promising shelf-life, as the *K*_i_ values determined after 6 months of storage at 40°C were not significantly altered when compared to those of newly prepared formulation. Moreover, the use of additives enhanced the enzyme activity (Srirangsan et al., 2010). Developments making the methodology amenable to implementation in microtiter plates and spectrophotometric quantification, thus allowing for high throughput, have been also presented (Goodrich and Balakireva, 2015).

**Figure 3:**
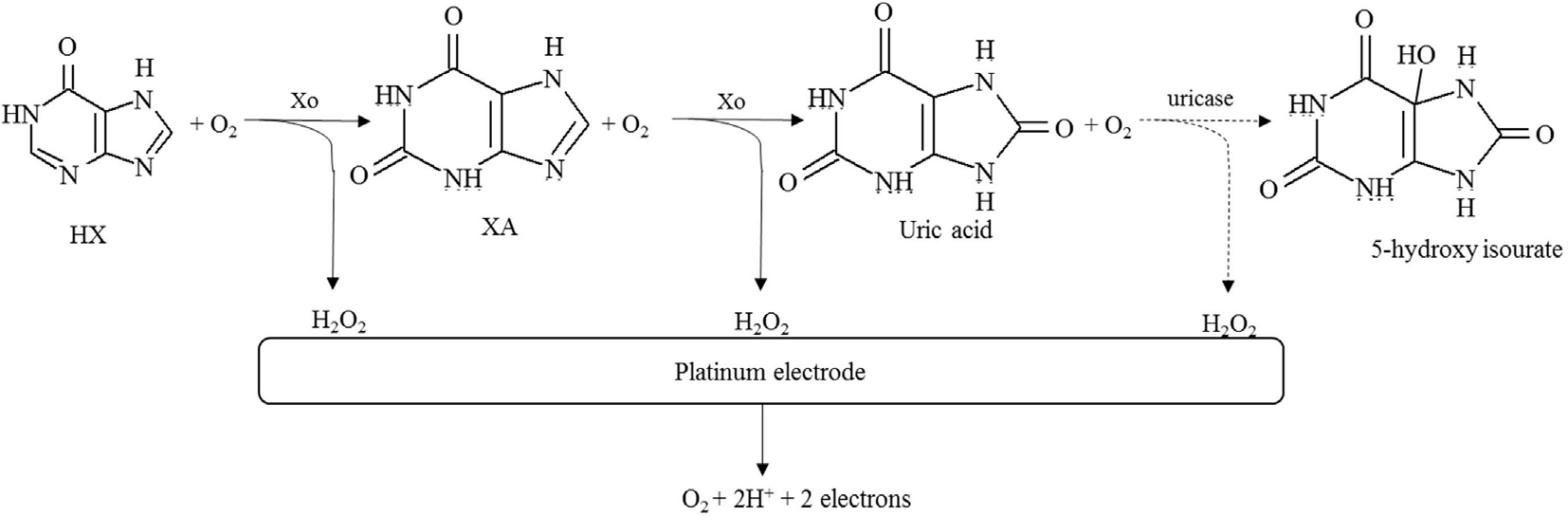
**Schematic diagram of the reactions involved in the quantification of hypoxanthine as an indicator of fish freshness**. The methodologies rely on the determination of hydrogen peroxide formed in consecutive reactions catalyzed by xanthine oxidase (solid lines) and eventually also uricase (dashed lines), combined with a platinum-based electrode. Further information and references as detailed in Table 3.

**Figure 4:**
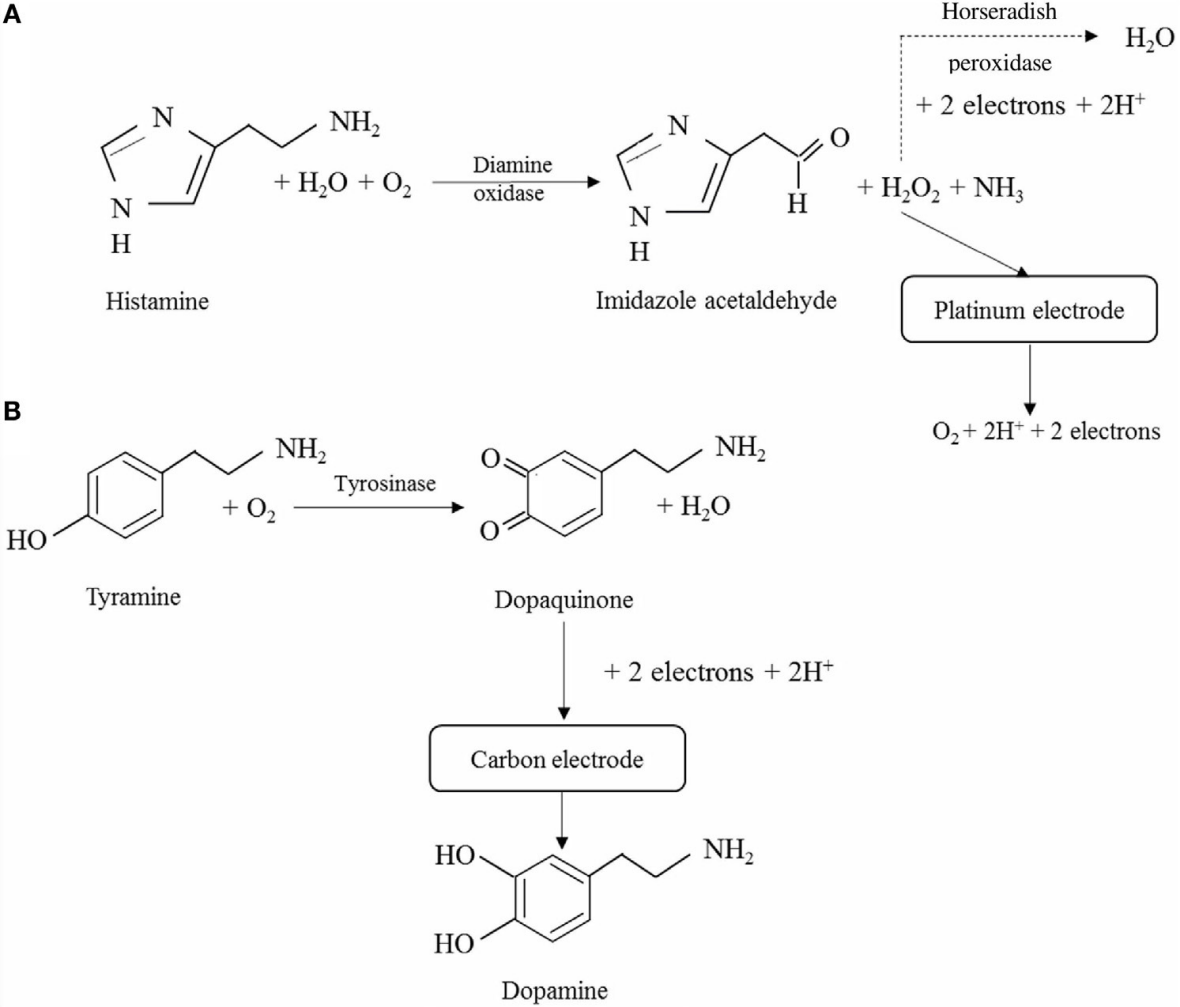
**Schematic diagrams of the reactions involved in the quantification of biogenic amines**. **(A)** Quantification of histamine based on hydrogen peroxide formation and either direct assessment of the latter in a platinum electrode (solid line, Apetrei and Apetrei, 2016) or through the use of horseradish peroxidase (dashed line, Pérez et al., 2013). **(B)** Quantification of tyrosine through oxidation with tyrosinase to dopaquinone and reduction of the latter to dopamine on the surface of a carbon electrode (Apetrei and Apetrei, 2015).

Assessment of fish and seafood freshness through the use of immobilized enzymes has relied on both multi-enzyme and single-enzyme systems, the former mostly aiming to determine several metabolites of ATP degradation and *K*_i_ values (Watanabe et al., 1984, 1986; Luong et al., 1989). Some of these multi-enzymes involved the flow injection analysis approach (Carsol and Mascini, 1998; Okuma and Watanabe, 2002). Still, enzymatic biosensors often rely on the quantification of HX using immobilized Xo, although detection of BAs has also been considered (Lawal and Adeloju, 2012a; Visciano et al., 2012). Immobilization strategies have been selected both aiming to allow for activity retention upon immobilization and enzyme stability and also to remove interferences form hydrogen peroxide, uric acid, or ascorbic acid that can be present in the sample for analysis (Aristoy et al., 2010; Lawal and Adeloju, 2012a). Specific details on the methods of immobilization and operation of recently developed biosensors are given in Table 3 and in Figures 3 and 4.

**Table 3:** **Recent examples of enzymatic biosensors developed for assessment of fish and seafood quality and freshness**.

Enzymes and immobilization method	Transducer	Comments	Reference
Xo immobilized over a copolymer of glycidyl methacrylate and vinylferrocene/multiwall carbon nanotubes for xanthine detection, based on H_2_O_2_ formed during substrate oxidation	Amperometric	Linear response to xanthine within 2–86 μM and LOD of 0.12 μM. Negligible interference from ascorbic and uric acid, sodium benzoate, and glucose.	Dervisevic et al. (2015a)
Xo covalently immobilized on a nanocomposite film constructed by embedding reduced expanded graphene oxide sheets decorated with iron oxide nanoparticles into poly(glycidyl methacrylate-*co*-vinylferrocene) phase. Xanthine detection as referred for Dervisevic et al., 2015a	Amperometric	Linear response to xanthine within 2–36 μM and LOD of 0.17 μM. Negligible interference from ascorbic and uric acid, sodium benzoate, and glucose. The biosensor retained 70% of the initial activity after 15 consecutive measurements.	Dervisevic et al. (2015b)
Xo immobilized electrostatically on a poly(vinyl ferrocenium perchlorate) matrix precipitated on a Pt surface for HX detection	Amperometric	Linear response to HX within 2.15 μM to 1.03 mM and LOD of 0.65 μM. A recovery of about 95% was observed as fish samples were spiked with 20 μM HX.	Bas et al. (2014)
Xo and ferrocene carboxylic acid entrapped into a polypyrrole film during galvanostatic polymerization film formation for HX detection	Potentiometric	Linear response to HX within 5–20 μM. Tested in fish samples with HX concentrations within 2.1 to 8.7 μmol/g. Decline in sensitivity after 5 days of storage	Lawal and Adeloju (2012b)
Xo and and uricase entrapped in a polypyrrole–polyvinyl sulfonate film by electrochemical polymerization in Pt formation for HX detection	Amperometric	Linear response to HX within 2.5–10 μM and 25 μM to 0.1 mM. The biosensor retained 74.5% of its initial performance after 20 assays and lost 44% of its initial performance after 33 days	Görgülü et al. (2013)
Xo and bovine serum albumin cross-linked with glutaraldehyde on membrane (Nafion)-coated surface of a Pt disk for HX detection	Amperometric	Linear response to HX within 2–185 μM. Tested in fish samples with HX concentrations within 0.877 and 16.38 μmol/g	Nakatani et al. (2005)
Diamine oxidase (Dox) immobilized over a carbon screen-printed electrode modified with a thick film of platinum nanoparticles, graphene and chitosan for histamine detection	Amperometric	Linear response to histamine within 0.1–300 μM and LOD of 0.0254 μM. The decay in biosensor response did not exceed 12.6% after 30 days of storage at 4°C. Interference from other biogenic amines, that is, cadaverine, tyramine, putrescine, and amino acids, was <10%	Apetrei and Apetrei (2016)
Dox and Hrp co-immobilized into a polysulfone–carbon nanotube–ferrocene membrane through phase inversion technique onto carbon screen-printed electrodes for histamine detection	Amperometric	Linear response to histamine within 0.3–20 μM and LOD of 0.17 μM. Matches to standard ELISA method results was reported for greater weever, mackerel and sardines.	Pérez et al. (2013)
Tyrosinase immobilized on carboxyl functionalized carbon nanotubes thick film of screen-printed electrodes by the casting method, and concomitant cross-linking with glutaraldehyde for tyramine analysis	Amperometric	Linear response to tyramine within 5–180 μM and LOD of 0.62 μM. Good reproducibility was observed for tyramine concentrations within 16.7 and 61.8 mg/kg. Close to 10% of recovery reported upon spiking with 40 mg/kg tyramine	Apetrei and Apetrei (2015)

## Conclusion

Fish and seafood industries have a key role as providers of healthy food. As an outcome of the increasing public awareness of the significance of a balanced diet to health, the demand for fish and seafood concomitantly grows, a trend that is foreseen to continue in the near future. Given the perishable nature of the products and their complexity, their effective processing and monitoring is a challenging task. Physical and chemical processes have been often the mainstay, with a minor contribution of endogenous enzymes. Advances in enzyme technology are turning the tide, as a result of a growing insight into the mechanisms of enzyme action, access to marine sources of enzymes, and improvements in heterologous expression, coupled with the need to minimize wastes and add value to byproducts that typically are often discarded. Within this, the enzymatic approach contributes significantly to overcome the environmental impact of traditional processes, thereby contributing to the implementation of sustainable and cost-effective processes. Moreover, enzyme technology can also contribute to the quality control of fish and seafood goods. It is a fact that the role of enzymes in fish and seafood industries lags behind other fields in food processing. Still, the combined developments in several complementary fields, such as heterologous expression of enzymes that can particularly allow for cost-effective production of some key enzymes, computational methods for predictive models, the ability to use the marine microbiota to provide new/improved enzymatic activities, enzyme formulations and immobilization methods, and materials science for enhanced operational stability or delivery systems, suggest that significant developments can be expected for enzyme applications in fish and seafood industries.

## Author Contributions

As a single author mauscript, all the work (concept, selection of papers/patents/thesis, design of the review, writing) is accounted to the one author.

## Conflict of Interest Statement

The author declares that the research was conducted in the absence of any commercial or financial relationships that could be construed as a potential conflict of interest.
